# Long-Lasting Effects of Sepsis on Circadian Rhythms in the Mouse

**DOI:** 10.1371/journal.pone.0047087

**Published:** 2012-10-11

**Authors:** Emma K. O'Callaghan, Sean T. Anderson, Paul N. Moynagh, Andrew N. Coogan

**Affiliations:** 1 Department of Psychology, National University of Ireland Maynooth, Maynooth, County Kildare, Republic of Ireland; 2 Institute of Immunology, National University of Ireland Maynooth, Maynooth, County Kildare, Republic of Ireland; Pennsylvania State University, United States of America

## Abstract

Daily patterns of activity and physiology are termed circadian rhythms and are driven primarily by an endogenous biological timekeeping system, with the master clock located in the suprachiasmatic nucleus. Previous studies have indicated reciprocal relationships between the circadian and the immune systems, although to date there have been only limited explorations of the long-term modulation of the circadian system by immune challenge, and it is to this question that we addressed ourselves in the current study. Sepsis was induced by peripheral treatment with lipopolysaccharide (5 mg/kg) and circadian rhythms were monitored following recovery. The basic parameters of circadian rhythmicity (free-running period and rhythm amplitude, entrainment to a light/dark cycle) were unaltered in post-septic animals compared to controls. Animals previously treated with LPS showed accelerated re-entrainment to a 6 hour advance of the light/dark cycle, and showed larger phase advances induced by photic stimulation in the late night phase. Photic induction of the immediate early genes c-FOS, EGR-1 and ARC was not altered, and neither was phase-shifting in response to treatment with the 5-HT-1a/7 agonist 8-OH-DPAT. Circadian expression of the clock gene product PER2 was altered in the suprachiasmatic nucleus of post-septic animals, and PER1 and PER2 expression patterns were altered also in the hippocampus. Examination of the suprachiasmatic nucleus 3 months after treatment with LPS showed persistent upregulation of the microglial markers CD-11b and F4/80, but no changes in the expression of various neuropeptides, cytokines, and intracellular signallers. The effects of sepsis on circadian rhythms does not seem to be driven by cell death, as 24 hours after LPS treatment there was no evidence for apoptosis in the suprachiasmatic nucleus as judged by TUNEL and cleaved-caspase 3 staining. Overall these data provide novel insight into how septic shock exerts chronic effects on the mammalian circadian system.

## Introduction

Circadian rhythms are recurring patterns in behaviour and physiology and many other parameters that repeat with periods of near 24 hrs under constant environmental conditions and are driven by an endogenous pacemaker [1]. In mammals, the suprachiasmatic nucleus (SCN) of the anterior ventral hypothalamus is the location of the master circadian oscillator which serves to coordinate the phase of other brain and peripheral circadian clocks [2], [3]. The molecular basis of circadian rhythm generation is reasonably well defined and involves a number of interlocking transcriptional loops of clock genes and post-translational regulation of their protein products [4]. This distributed circadian systems serves to impose a daily temporal regulation on numerous physiological and psychological processes, such as metabolism, immune function and cognition, and circadian rhythm disruption is associated with a number of pathological states [3], [5], [6].

An area in chronobiology that merits investigation is the extent to which circadian and immune systems influence each other [7]. There is emerging evidence regarding the effects of the circadian system on immune function and how circadian disruption impacts negatively upon immune processes [8], [9], [10]. Evidence regarding the effects of the immune system on the circadian system is sparser, although it is also increasingly evident that immune processes can modulate central and peripheral circadian clocks [7]. The SCN is shown to express immune mediators and their signalling intermediates [11], [12], [13], [14]. Recently, a number of studies have highlighted the ability of immune signallers to impact upon central timekeeping processes. Direct administration of immune signals such as LPS [15] and cytokines [13], [16] has been shown to phase shift circadian rhythms. The pro-inflammatory cytokines tumour necrosis (TNF)-α [17] and interferon (IFN)-γ [18] also induce changes in the electrophysiological properties of SCN neurones. Further, immune stimulation with peripheral LPS induces SCN expression of the immediate early gene c-Fos and the p65 subunit of NF-κB, showing that cellular activity in the SCN is modulated by immune status [11].

To date work has focussed on the acute impact of immune challenge/immunomodulators on circadian processes, but the long-lasting effects of immune challenge on circadian rhythms has so far not been systematically investigated. However, clinical data indicates that there are long-lasting effects of sepsis (a profound pathophysiological state elicited by uncontrolled infection and subsequent massive systemic inflammation associated with very significant mortality [19]), with up to 50% of sepsis survivors suffering from post-septic encephalopathy, characterised by long-lasting cognitive difficulties [20]. In animal models sepsis can be induced by a number of methods, the most commonly used ones being cecal ligation and puncture (CLP) or by peripheral challenge with a high dose of LPS [21]. In mice it has been shown that treatment with a single septic dose of LPS induces a long-lasting central neuroinflammation [22] and cognitive changes [23]. Palomba and Bentivoglio [24] showed that weekly injections with LPS altered the photic-responsiveness of the SCN as measured by c-FOS induction, indicating that sepsis may impact on the master circadian clock. Furthermore, chronic infection that leads to ongoing neuroinflammation, such as trypansomiasis, is also associated with altered circadian function [25], [26]. Given the widespread influence of the circadian timing system on physiological and psychological domains, and therefore the possibility that circadian modulation may be an important factor in mediating the long-term effects of sepsis, we set out to examine the long-term impact of sepsis on circadian rhythms in the mouse. We have used a single septic dose of LPS in mice and monitored behavioural circadian rhythms, patterns of clock gene product expression and long-lasting changes in inflammatory markers in the SCN.

## Materials and Methods

### Animals

All experiments were carried out on male C57Bl/6 mice (Charles River, Kent, UK), which were between 8 and 10 weeks old at the start of each experiment. All procedures were approved by the Research Ethics Committee, National University of Ireland Maynooth, and were licensed by the Department of Health and Children, Ireland as per the Cruelty to Animals Act (1876, amended 2002 and 2005). Throughout all of the experiments food and water was available *ad libitum*, temperature was 21±1°C and humidity was 50±10%. For the purpose of behavioural monitoring, animals were individually housed in polypropylene cages (33 cm long×15 cm wide×13 cm high) equipped with steel running wheels (11.5 cm diameter). The cages were fitted with micro-switches connected to a data acquisition system computer using the Chronobiology Kit by Stanford Systems (Santa Cruz, California) for recording of daily rhythms of locomotor activity. The light source was standard fluorescent light bulbs with an average illuminance of 210 lux at cage level. Animals were maintained on a 12∶12 light dark (LD) photoperiod for at least 2 weeks prior to any experimentation.

### Treatment

Sepsis was induced with an intraperitoneal (i.p.) injection of 5 mg/kg LPS (serotype 0111.B4, Sigma Ireland) which occurred between zeitgeber time (ZT) 6 and ZT8, where ZT0 is defined as the time of lights on. Control animals received the same volume (0.12 ml) i.p. of sterile saline. Prior to any photic or other circadian manipulation, animals were allowed to recover for at least two weeks after the LPS treatment. We encountered a rate of mortality and significant moribundity requiring euthanasia of approximately 10% across all experiments following sepsis induction.

### Jet-lag experiment

Animals were entrained to a 12∶12 L∶D cycle for 14 days before receiving an i.p. injection of LPS (n = 11) or saline (n = 12). 18–20 days following the treatment the light dark cycle was advanced by 6 hours (by means of a “short day” when lights on occurs 6 hours earlier than normal, and so the lights on period for that “day” is only 6 hours), and this schedule was maintained for 14 days. After this the L∶D cycle was delayed by 6 hours (by means of a “long day” in which the onset of darkness is delayed by 6 hours, and therefore the animals experience an 18 h day), and animals held under this cycle for a further 14 days. Following this animals were released either into constant darkness (DD) or constant light (LL) for a further 14 days to assess free-running circadian parameters. Free running rhythm period and amplitude were assessed via chi-square periodograms run on the Kit Analyze software of the Chronobiology Kit. After this period animals were re-entrained to a 12∶12 L∶D cycle for 2 weeks prior to being perfused for immunohistochemistry. To assess entrainment to the shifted L∶D cycle times of activity onset for each mouse were assessed for each daily cycle, with the day of the change in light onset being defined as the first day of the shifted cycle. Re-entrainment was deemed to have occurred when the onset of activity occurs within ±20 minutes of the time of lights out. Rates of change in activity onset for the 7 days following the phase-shift was also assessed by mixed between-within groups ANOVA with activity onset time as the dependent variable.

### Photic phase response curve

For this experiment LPS-treated (n = 14) and saline-treated (n = 16) animals were placed into DD 2 weeks after treatment and allowed to free-run for 2 weeks. Animals then received 30 minute light pulses (∼200 lux) at different points of the circadian cycle. Initially pulses were aimed at CT15 and CT22, as these are the circadian phases associated with maximal phase delays and advances respectively in mice [27], but after this animals received light pulses across the circadian cycle, with each animal receiving a maximum of 5 light pulses. Following each light pulse animals activity pattern in DD was followed for 14 days before the next pulse was given. Phase-shifts were assessed with the line of best fit method, using the onsets of activity for 7 cycles prior to the light-pulse and the onsets for 7 cycles starting 4 days after the light pulse to minimise interference from transients. Actograms were rated by three independent raters.

### Photic-induction of immediate early genes in the SCN

Following the phase-response curve experiment, animals were placed into a 12∶12 L∶D cycle for two weeks, prior to being placed into DD for 2 cycles. They then received a 30 minute light pulse at either CT15 or CT22 and 1 hour after the start of the pulse were sacrificed, their brains removed and immersion fixed in 4% PFA for immunohistochemical processing for the immediate early gene products c-FOS, EGR-1 and ARC.

### Non-photic phase shifting

In order to test non-photic response, mice (n = 7) were treated with LPS (5 mg/kg) or with saline (n = 7) under LD cycle conditions, allowed 2 weeks for recovery and then placed into DD for a further two weeks. These animals then received an i.p. injection of the serotoninergic agonist 8OH-DPAT (5 mg/kg i.p.,Tocris Bioscience, Bristol, UK) at CT6, and were maintained in DD for a further two weeks to allow for assessment of phase-shifts. This dose of 8-OH-DPAT has previously been shown to induce maximal phase-shifts in mice [28].

### Entrainment to skeleton photoperiod

In order to delineate entrainment from any masking effects of the light dark cycle, LPS treated (n = 7) and saline (n = 9) animals were exposed to a 12∶12 L∶D cycle for two weeks. The light dark cycle was then shortened to a skeleton photoperiod whereby light occurred during only the first and last hour of the previous light cycle followed by the original dark phase (1L∶10D∶1L∶12D). Following 3 weeks in these conditions the light∶dark cycle was then made a half skeleton photoperiod where the first light phase was then removed so that the only light phase occurred during the final hour of the original light cycle (IL∶23D). Entrainment was assessed by comparing the time of activity onset to the time of the skeleton photoperiod for each 24 hour cycle.

### Assessment of circadian clock gene expression

In order to assess the rhythmic expression of circadian clock gene product expression, group housed animals were treated with either LPS (5 mg/kg) or saline and allowed to recover for 1 month. Following this they were placed in DD for two cycles and on the third cycle were sampled every four hours across the twenty four cycle in dim red light. Animals were intracardially perfused as previously described [11], [12] following terminal anaesthesia induced by sodium pentobarbital given i.p. under dim red right (<1 lux). Group size was n = 3 or 4 per timepoint per treatment group. Brains were then processed by immonohistochemistry for PER1, PER2, CLOCK and c-FOS (details of the antibodies given in Table S1). The use of these antibodies has been detailed in a number of previous studies [29], [30], [31] and these produce staining patterns very similar to those seen in a recent study validating a panel of clock gene product antibodies [32].

### Assessment of acute effects of LPS on SCN

In order to assess whether a septic dose of LPS would induce signs of apoptosis animals were injected with either LPS (5 mg/kg) or saline (n = 4 for both groups) and twenty four hours later, still in the acute phase of sepsis, animals were perfused and their brains removed. These were then processed for TUNEL staining (DeadEnd Fluorometric TUNEL staining kit, Promega, UK according to manufacturer's instructions) and for cleaved-caspase 3 staining. Further sections were stained for the microglial marker F4/80, TNF-α, NOS2, EGR-1 and ARC. Details of antibodies used are in Table S1.

### Assessment of chronic effects of LPS on SCN neurochemistry

To assess the long-term effects of LPS-induced sepsis on the SCN, at the termination of the circadian activity monitoring experiments detailed above, animals were housed in 12∶12 L∶D for two weeks and then animals were terminally anaesthetized with 0.1 ml sodium pentobarbitone and perfused transcardially with 0.9% saline followed by 4% paraformaldehyde in 0.1 M Phosphate Buffer. Brains were carefully removed, post-fixed overnight and were cryoprotected in 30% Sucrose (Sigma) for 24 hrs. 30 µm thick serial coronal sections were cut throughout the rostrocaudal extent of the SCN on a freezing stage microtome (Leica). All sections through the SCN were collected and divided in 4 series, obtaining 5 or 6 sections through the SCN in each series. Brains were then processed by immunohistochemistry for a number of markers of glia (F4/80 and CD11-b as microglial markers, GFAP as an astrocytic markers), for pro-inflammatory cytokines (IL-1β, TNF-α), for immediate early gene (IEG) expression (ARC, EGR-1), for signallers known to be induced by neuroimmune processes (NOS2, P65 NF-κB subunit, phosphorylated (p)-IκK, p-IκB) and for neuropeptides expressed in the SCN (VIP and AVP). Details of all antibodies used are in Table S1. Immunohistochemistry was by a standard Avidin-Biotin Complex/Nickel DAB colourometric protocol [11], [12]. Where possible, sections for the same antigen from different groups were reacted in parallel, and when this was not possible development of immunostaining was standardised between runs (eg. same amount of time developing in Ni-DAB) to minimise as much as possible inter-run variability. For analysis of immunostained sections photomicrographs of the mid-rostrocaudal level of the SCN were taken using a digital camera connected to an Olympus BX-51 light microscope equipped with an image analysis digital system (ImageJ 1.43, NIH, USA). Between 3 and 6 images were evaluated for each individual animal and region to give a mean value for each animal. Immunoreactive cells in each region of interest were quantified using either image analysis software or number of immunoreactive cells quantified by an observer (for clock gene proteins and immediate early genes). The observer was blinded to the experimental procedure during optical density measurements or quantification of immunoreactive cells per SCN. The difference in integrated optical density (IOD) of antibody immunosignal in the SCN or the number of immunoreactive cells that displayed clear nuclear staining in the SCN of all animal groups was evaluated for quantitative analysis. The light intensity was kept constant while all measurements were taken to standardize IOD measurements for analysis. A previously described method whereby the image was binarised for analysis was used for integrated optical density measurements [33], [34].

### Statistical Analysis

All data values given are means ± SEM. Inferential statistical analysis was via factorial mixed between-within groups ANOVAs and t-tests as appropriate. P<0.05 was deemed statistically significant. Where multiple comparisons were carried out the appropriate Bonferroni correction was applied. For analysis of circadian patterns of clock gene protein expression the CircWave 1.4 analyses software (Dr. R. Hut, http://www.euclock.org), which employs a forward linear harmonic regression to calculate the profile of the wave with a 24 hr period, was used. A 24 hour rhythm was confirmed if the null amplitude hypothesis was rejected from an F test producing a significant value p<0.05.

## Results

### Effects of sepsis on behavioural circadian rhythms

**Figure 1:**
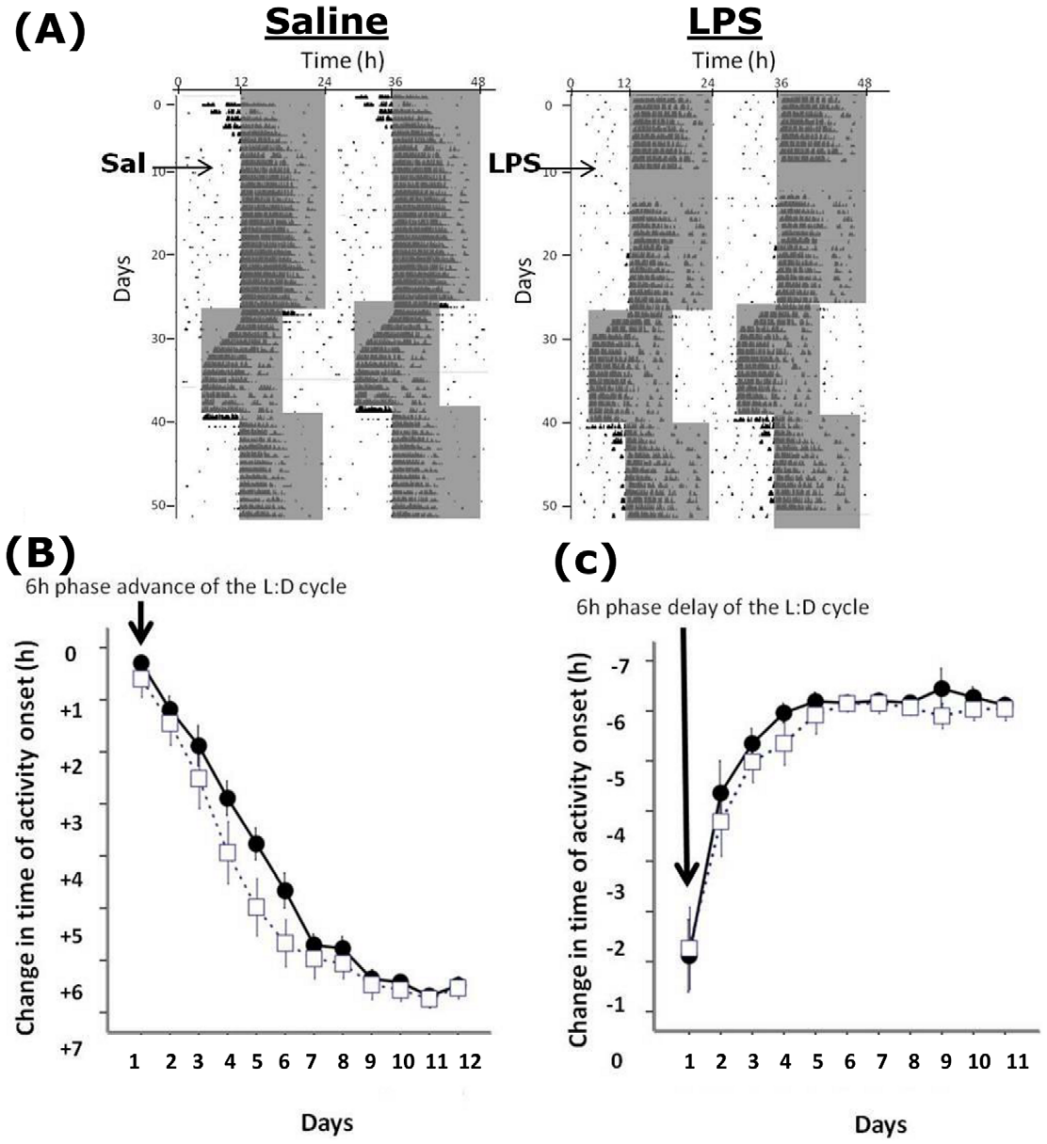
Entrainment to L∶D cycles and re-entrainment to shifts in the L∶D cycle. (A) Sample double plotted actograms from a control and a LPS treated animal. The shaded area is the dark phase of the L∶D cycle. Note the suppression of wheel running activity for a number of cycles following LPS treatment, but when activity resumed it did so at the expected phase. Animals were then exposed to a 6 hour advance of the L∶D cycle, and then a 6 hour delay of the L∶D cycle. (B) and (C) show the rates of re-entrainment of the control and LPS animals to the shifted cycles. Note the more rapid adjustment of the activity onsets in the LPS group compared to the controls following the advance (C) but not the delay (D).

**Figure 2:**
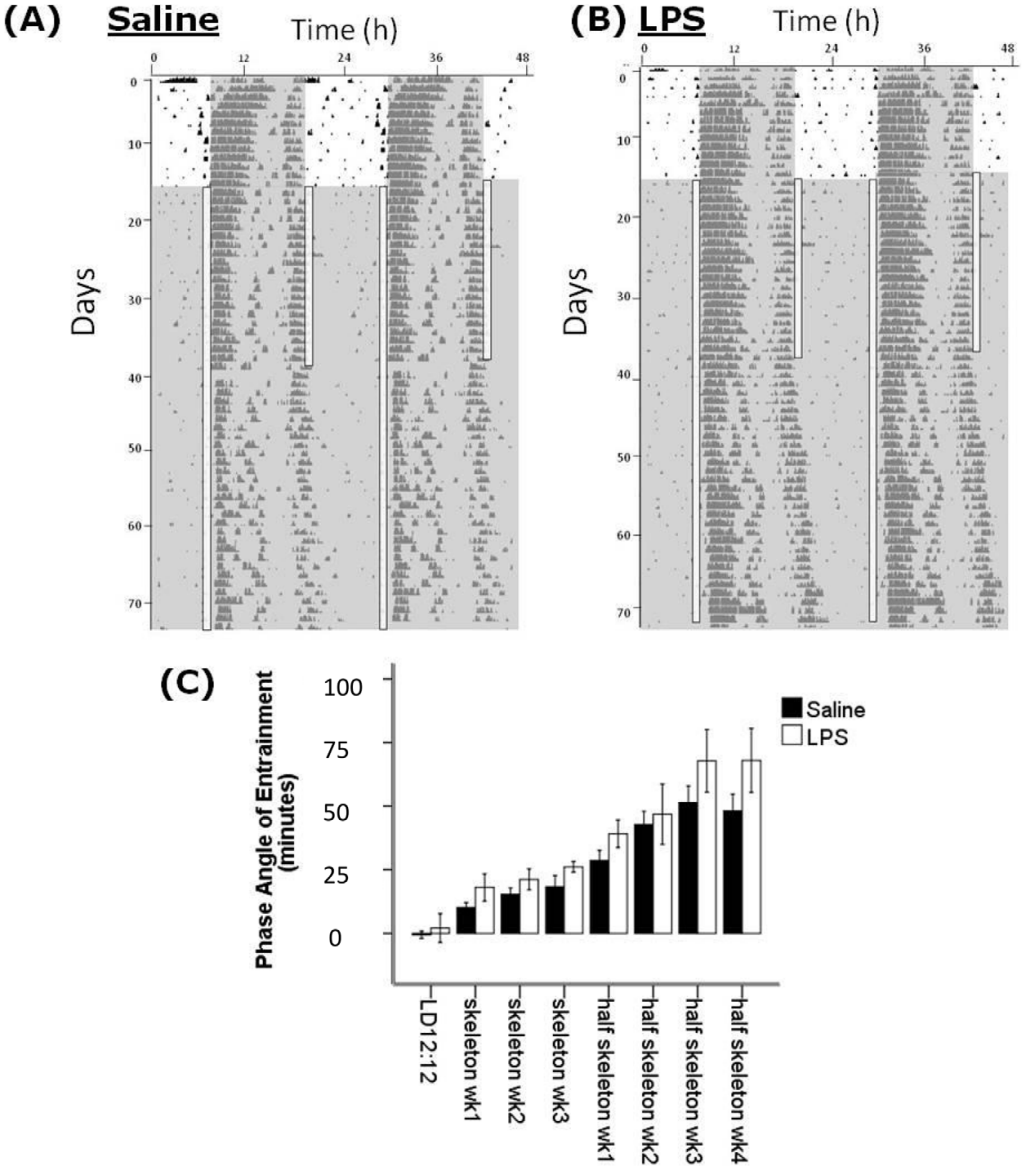
Entrainment to skeleton photoperiods. (A) and (B) are sample double plotted actograms from a control and a LPS-treated animal respectively showing entrainment to a 12∶12 L∶D cycle, then a 1∶10∶1∶12 skeleton photoperiod and subsequently a 1∶11∶12 half skeleton photoperiod. (C) shows the average phase-angles of entrainment per week of the experiment for the control and LPS groups. There are no significant groupwise differences.

Animals treated with LPS did not display any wheel running activity for 2–4 days after the injection. Upon resumption of the wheel running activity, the circadian pattern of activity resumed at the expected phase, and there was no alterations in the relationship between the activity onset and the onset of the dark phase of the L∶D cycle (Figure 1). There were also no differences between the LPS-treated animals and saline controls in overall activity levels (as measured by wheel revolutions per hour), in the amplitude of the locomotor rhythm in LD, LL or DD, or in the free running period in DD or LL (Table 1). Therefore on a gross level, the basic parameters of the circadian locomotor rhythm in post-septic animals appeared normal. When animals that had previously received LPS were exposed to a 6 hour advance of the L∶D cycle, re-entrainment to the new cycle was faster than in saline control animals, with a mixed factorial ANOVA for time of onset of activity for the 7 days following the advance showing a time x treatment interaction (F_15,6_ = 4.47, P<0.01; Figure 1). The time to entrain to the new cycle was 5.09±0.48 days for post-septic animals vs. 7.08±0.43 days for controls (P<0.01). When these animals were then exposed to a 6 hour delay of the L∶D cycle there were no significant effects of treatment from the ANOVA, and the mean time to re-entrain was 3.1±0.2 days for post-septic animals vs. 3.2±0.3 days for controls (P>0.05). To further examine whether the apparently normal pattern of activity under a L∶D cycle reflects appropriate circadian entrainment, or whether underlying circadian abnormalities were being masked by the L∶D cycle, we examined the entrainment of a group of animals to first a 1∶10∶1∶12 skeleton photoperiod and then to a 1∶23 half skeleton photoperiod, and examined the phase angle of entrainment (Figure 2). We observed no significant differences in the entrainment of the post-LPS animals when compared to controls at any point during this experiment, indicating that true entrainment was normal in the post-septic animals.

**Table 1:** Details of the mean (+SEM) circadian parameters for control and previously LPS-treated animals.

	Control (n = 8)	LPS (n = 8)
Rhythm Amplitude in LD	865.93+/−42.3	885.67+/−38.2 (n.s.)
Free running period in LL (h)	25.13+/−0.10	24.99+/−0.19 (n.s.)
Rhythm Amplitude in LL	748.83+/−85.53	708.24+/−76.83 (n.s.)
Free running period in DD (h)	23.73+/−0.14	23.6+/−0.11 (n.s.)
Rhythm Amplitude in DD	1357.48+/−232.7	1382.7+/−227.7 (n.s.)
Wheel revolutions per day	9055+/−3641.73	7676.48+/−2095.7 (n.s.)

There were no significant differences (n.s.) between the groups in terms of rhythm period, rhythm amplitude or amount of wheel-running acitivity.

**Figure 3:**
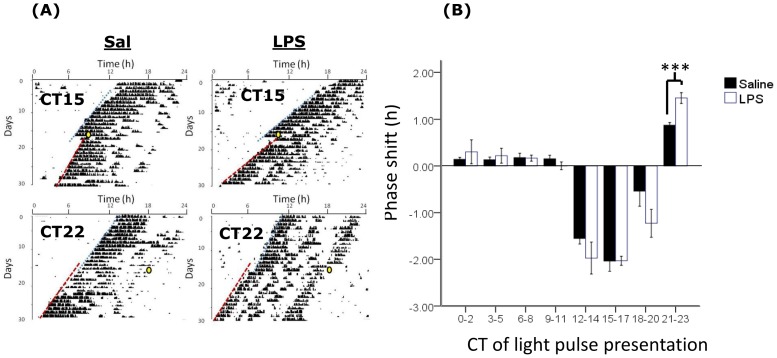
Photic phase-response curve in saline and LPS treated animals. (A) Sample single plotted actograms of control and LPS treated animals free running in DD and exposed to light pulses at CT15 and CT22 (indicated by yellow circles, with lines indicating lines of best-fit through activity onsets). (B) Magnitude of photic phase shifts induced by light pulses at different circadian times (CT) across the 24 h cycle. Responses were collated into 3 hour bins according to the time of presentation of the light pulses. ***<0.001.

**Figure 4:**
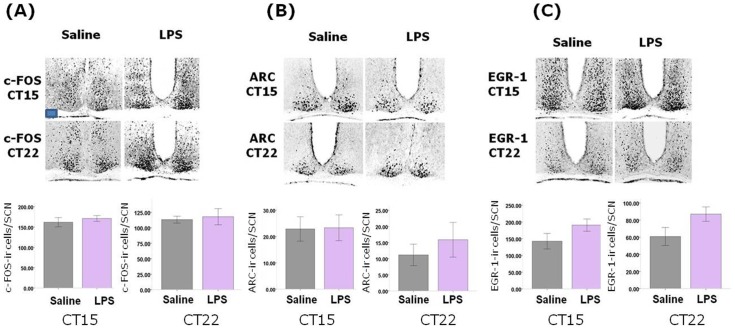
Effects of previous sepsis on light induction of IEGs in the SCN. Light pulses were delivered at CT15 and CT22 in saline and LPS animals and levels of (A) c-FOS, (B) ARC and (C) EGR-1 were assessed in the SCN. There were no significant differences in any of these levels across the control and sepsis groups. n = 4 for each group at each CT. Scale bar = 100 µm.

We then examined the resetting of circadian rhythms to photic stimulation at various times across the circadian cycle by constructing phase-response curves for both the post-septic and control animals. When the phase-shifts from these experiments was analysed by 2-way factorial ANOVA we noted a significant time of light pulse x treatment interaction term (F_110,7_ = 2.17, P<0.05), with a larger magnitude phase shift elicited in the post-septic animals following photic stimulation in the CT22-24 bin than in controls (P<0.001; Figure 3). At all other points in the cycle there were no groupwise differences in the magnitude of photic phase shifts. To further investigate the effects of photic stimulation on circadian function we examined the expression of the immediate early genes c-FOS, ARC and EGR-1 in the SCN following photic stimulation during either the delay portion of the PRC (CT15) or the advance portion (CT22). We found no differences in the levels of each of these antigens at either phase between the saline and post-septic animals, indicating that SCN cellular activation elicited by photic stimulation is unaltered by previous sepsis (Figure 4).

**Figure 5:**
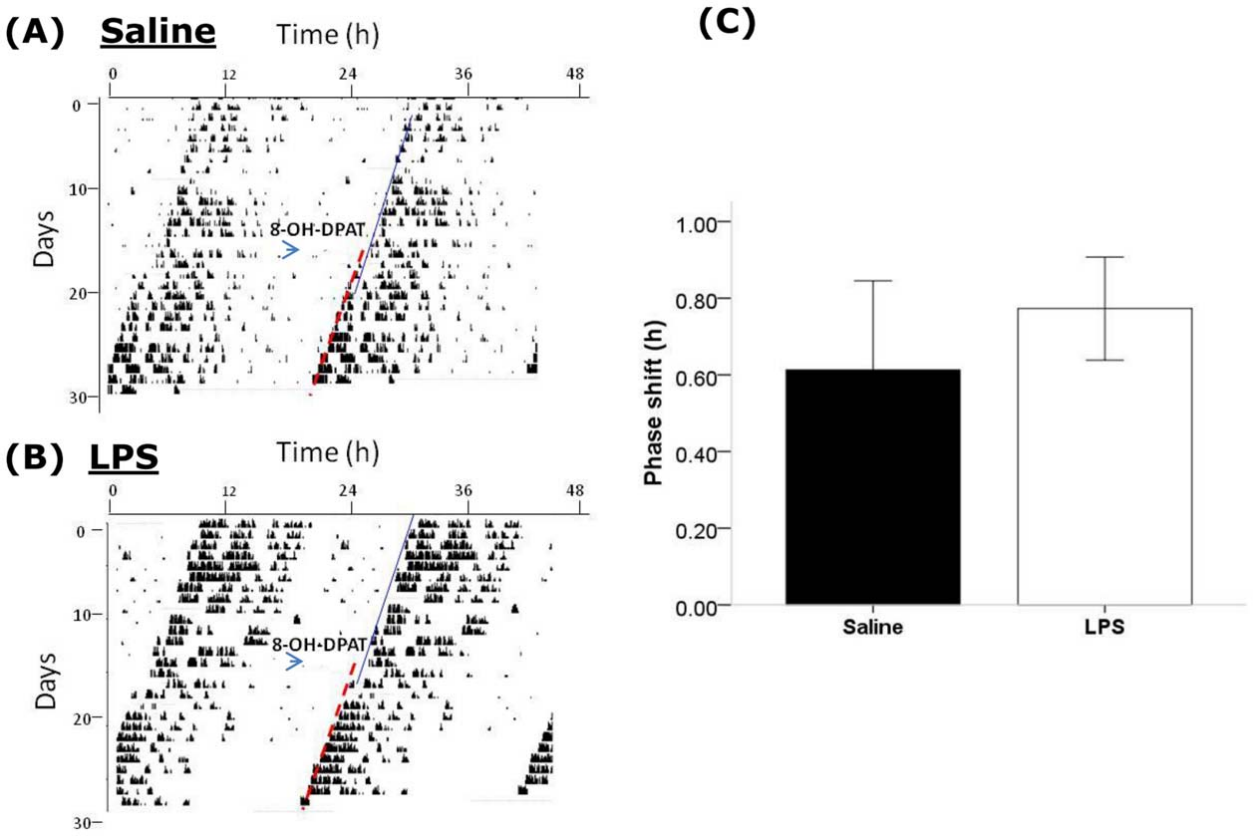
Effects of previous sepsis on non-photic phase-shifting via a serotonergic agonist. Sample double plotted actograms of a control animal treated with 8-OH-DPAT at CT6 (A) and an animal previously treated with LPS and then treated with 8-OH-DPAT at CT6 (B). In both cases 8-OH-DPAT induces a modest phase advance, but the magnitude of this phase shift did not vary between groups (C).

In order to examine whether the changes in phase advances observed in post-septic animals in both the jet-lag experiment and the PRC experiment may be generalised to altered phase-advancing following any type of stimulus, we examined the phase shifts elicited by a non-photic stimulus, application of the 5-HT1a/7 agonist 8-OH-DPAT at CT6. In control animals, 8-OH-DPAT treatment lead to a modest phase advance (0.62±0.23 h; Figure 5) which did not differ significantly from that induced in post-septic animals (0.77±0.13 h, P>0.05). Therefore, the previous induction of sepsis by LPS did not seem to alter the subsequent responses of mice to 8-OH-DPAT administered during the subjective day.

### Long-term effects of sepsis on clock gene protein expression

**Figure 6:**
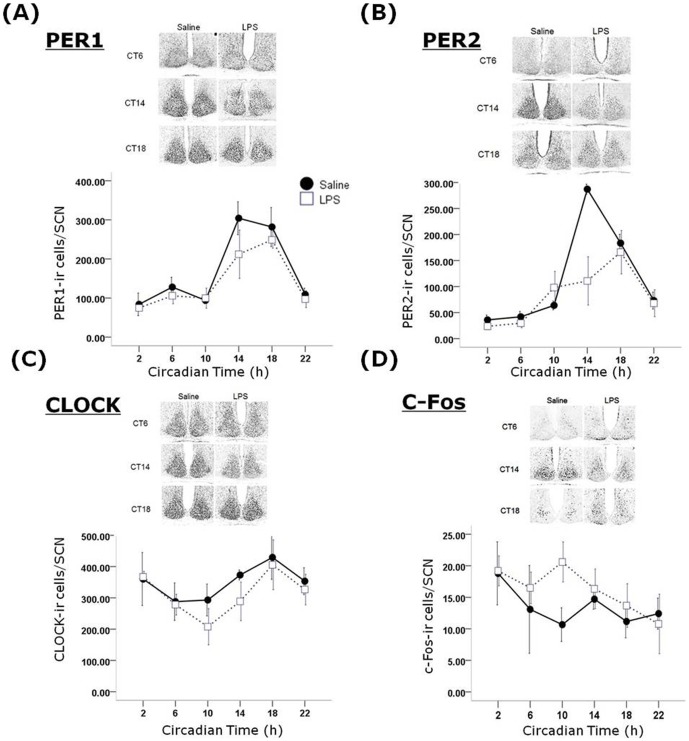
Effects of previous sepsis on clock gene product expression in the SCN. Expression patterns of PER1 (A), PER2 (B), CLOCK (C) and c-FOS (D) were examined in the SCN of animals treated one month previous with either saline or LPS and then sampled every 4 hours across the 24 hour cycle in DD. n = 4–5 per timepoint per group. Note the significant change of expression of PER2 in the SCN of LPS treated animals.

**Figure 7:**
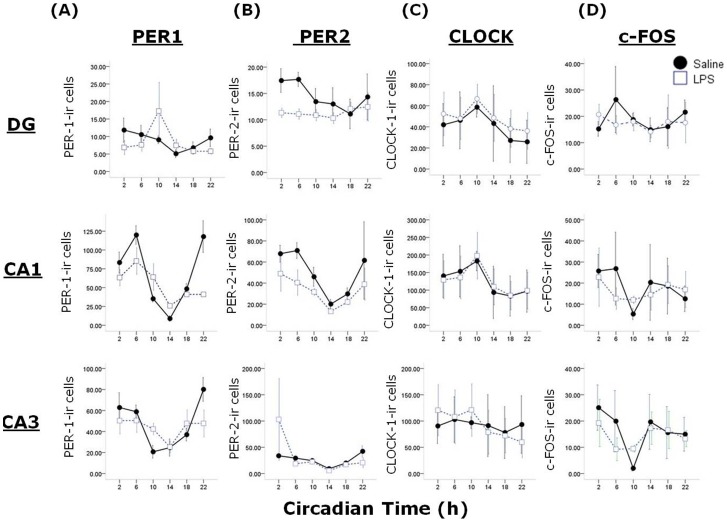
Effects of previous sepsis on clock gene product expression in the hippocampal subfields dentate gyrus (DG), CA1 and CA3. Expression patterns of PER1 (A), PER2 (B), CLOCK (C) and c-FOS (D) were examined in the hippocampus of animals treated one month previous with either saline or LPS and then sampled every 4 hours across the 24 hour cycle in DD. Note the significant change of expression of PER1 in the CA1 and PER2 in the DG and CA1 of animals previously treated with LPS.

As behavioural circadian rhythms are known to be driven by molecular oscillations in the SCN and other clock sites, we examined the rhythmic expression of three clock gene protein products (PER1, PER2, CLOCK) and c-FOS in the SCN and the hippocampus across the circadian cycle. In the SCN we observed a main effect of treatment (F_1,42_ = 4.72, P<0.05) and a significant time x treatment interaction (F_5,42_ = 4.26, P<0.01; Figure 6) on PER2 expression. Co-sinor analysis of PER2 expression in the SCN reveals that the acrophase of expression appears to be shifted from CT15.9 in control animals to CT17.5 in post-septic animals (although this may be due to decreased expression at CT18 in post-LPS animals, rather than a true phase shift), and that the percentage of variance in PER2 expression accounted for by a 24 hour co-sine wave is reduced from 91% in controls to 38% in post-septic animals. There were no significant treatment or interaction effects detected for PER1, CLOCK or c-FOS expression in the SCN. As other central sites show rhythmic clock gene protein expression, we also examined PER1, PER2, CLOCK and c-FOS expression in the hippocampal subfields dentate gyrus (DG), CA3 and CA1 (Figure 7). We choose the hippocampus as a sub-cortical region with well known roles in cognition that has also been shown in a number of studies to show circadian rhythmicity in clock gene expression, which in turn appears to underpin the cognitive and behavioural function of the hippocampus (5). In the CA1 there was a main effect of treatment (F_1,36 = _5.2, P<0.05) and a time x treatment interaction on PER1 (F_5,36 = _5.2, P<0.01) expression, and a main effect of treatment on PER2 (F_1,36 = _4.47, P<0.05), but no effects on CLOCK or c-FOS expression. In the CA3 there were no significant effects of treatment or treatment x time interactions for any of the four antigens, and in the DG there was a main effect of treatment on PER2 expression (F_1,36 = _5.2, P<0.05) but there were no other significant effects in the DG. It is worth noting there was some large variation in the clock gene product expression in the hippocampus, especially in LPS-treated animals (eg. PER1 at CT10 in the DG).

### Neuroinflammation and other long-term changes in the SCN

**Figure 8:**
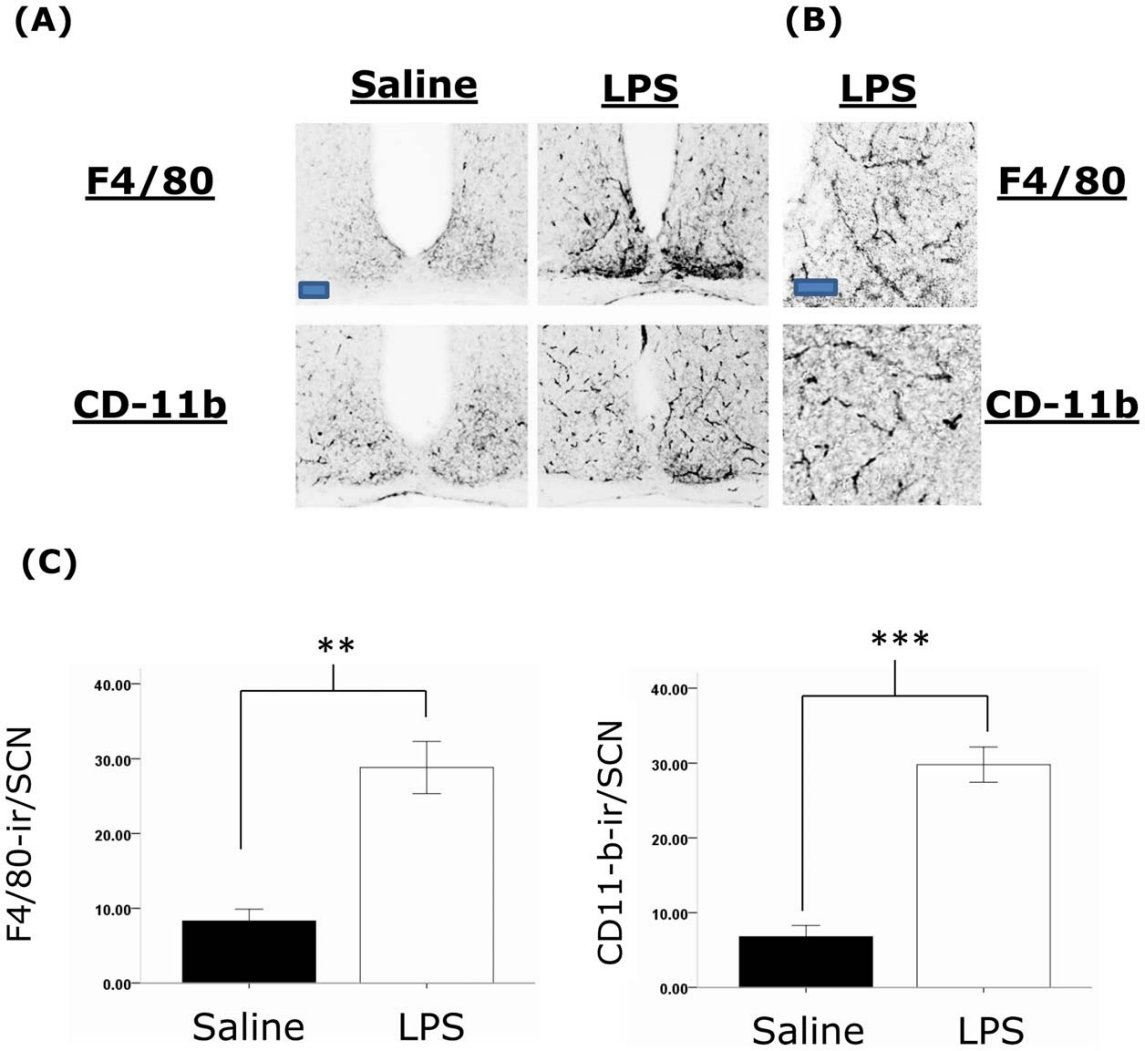
Previous sepsis results in a long-lasting upregulation of the microglial markers F4/80 and CD-11b in the SCN. (A) Representative photomicrographs of F4/80 and CD-11b expression in the SCN of animals treated 3 months previously with either saline or LPS. Animals were sampled mid subjective day (ZT5-8) in a 12∶12 L∶D cycle. Note the significant upregulation of both antigens in the SCN, and also the ramified morphology of the cells in the post-septic SCN (scale bar = 100 µm). These are shown in higher magnification in (B) in SCNs from LPS treated animals (scale bar = 50 µm). (C) Quantification of levels of immunoreactivity for F4/80 (n = 4 for both groups) and CD-11b (n = 6 for both groups) in the SCN in the control and LPS treated groups. ** P<0.01; *** P<0.001.

**Figure 9:**
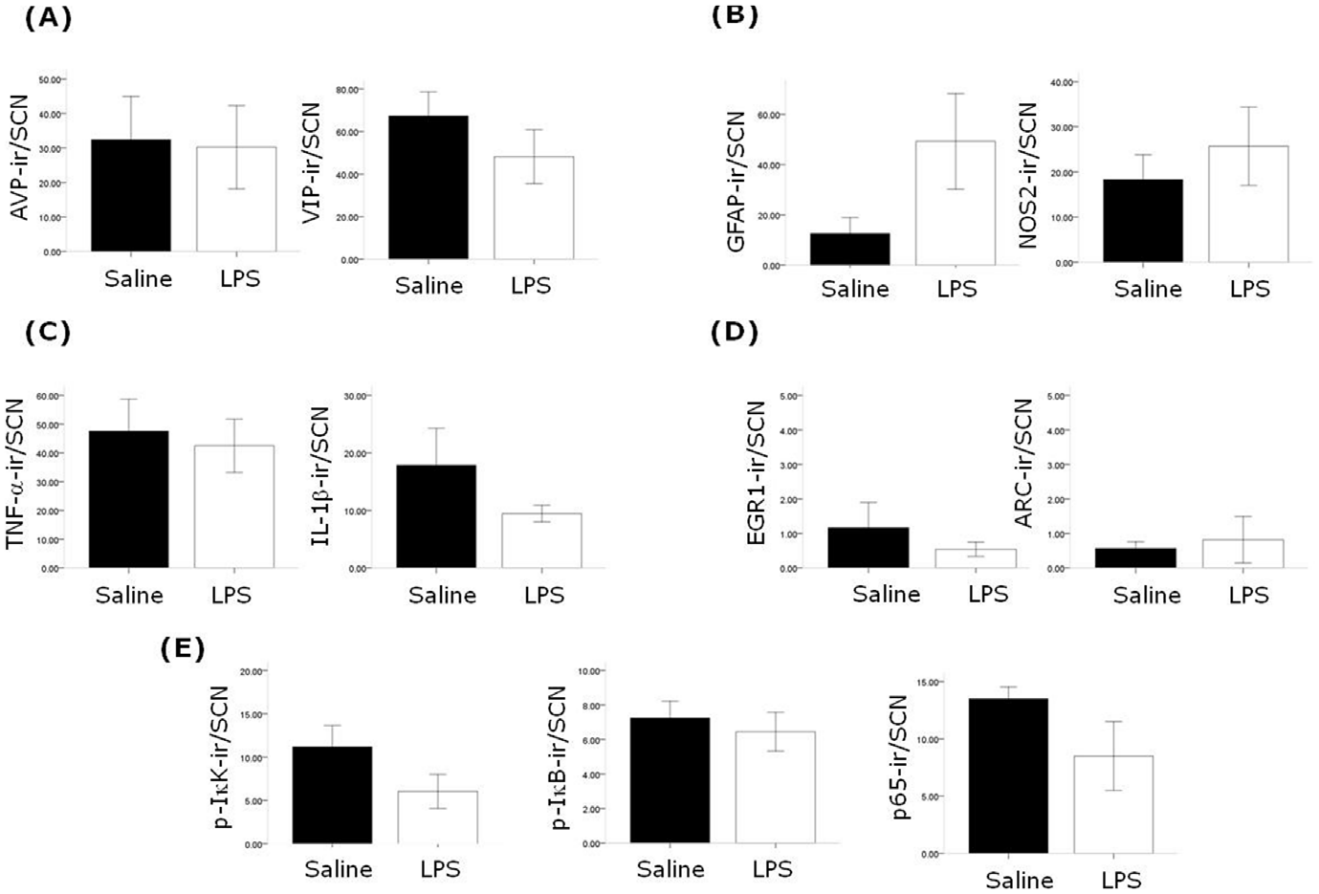
Chronic effects of sepsis on SCN neurochemistry. Animals were sampled mid subjective day (ZT5-8) in a 12∶12 L∶D cycle. The neuropeptides AVP and VIP (A) were unaltered in the SCN (n = 7 for saline and LPS groups), 3 months after treatment with LPS compared to saline treated controls, as were GFAP and NOS2 (B; n = 7 for both saline and LPS groups; there was considerable variability of GFAP staining in the LPS group), the pro-inflammatory cytokines TNF-α and IL-1β (C; n = 5–6 for the saline and LPS groups), the IEGs EGR-1 and ARC (D; n = 4 for the saline and LPS groups) and components of the NF-κB signalling pathway p-IκK, p-IκB and p65 (E; n = 5 for the saline and LPS groups).

**Figure 10:**
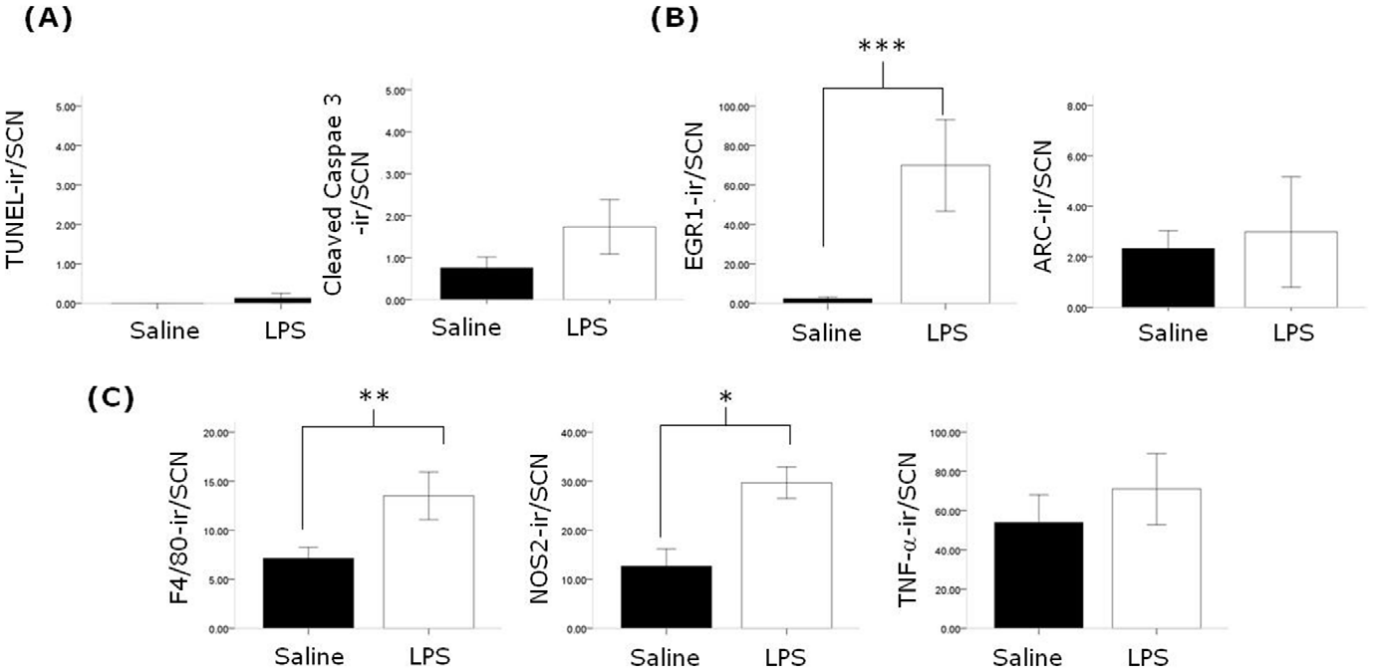
Acute effects of LPS treatment on SCN. Animals were treated with either saline or LPS and 24 hours later sampled. There was no increase in the markers for apoptosis TUNEL and cleaved-caspase 3 (A; n = 4–5 for the saline and acute LPS groups), there was a significant increase in EGR-1 but not ARC (B; n = 5 for the saline and acute LPS groups), and whilst F4/80 and NOS2 immunoreactivity in the SCN was significantly elevated in the LPS-treated animals, TNF-α was not (n = 4–5 for the saline and acute LPS groups). *** P<0.001, ** P<0.01, * P<0.05.

To examine whether the alterations in behavioural and clock gene protein rhythms described above are accompanied by changes in neuroimmune factors in the SCN, we examined the expression of a number of factors three months following the induction of sepsis in animals sampled under 12∶12 L∶D cycles in the middle of the lights on phase (ZT5-8). In post-septic animals we observe significant upregulation of the microglial markers F4/80 and CD-11b in the SCN (Figure 8). It should be noted that the morphology of the cells expressing F/80 and CD-11b appeared to be ramified, suggesting that these microglia were not in an active state. The astrocytic marker GFAP did not show a significant upregulation in the SCN, and neither were there significant changes in the expression of TNF-α, IL-1β, NOS2, or components of the NF-κB signalling pathway (Figure 9). Further we did not observe changes in the expression of the SCN neuropeptides AVP and VIP, nor of EGR-1 and ARC (Figure 9). In order to examine the changes that may occur in the SCN during the acute phase of sepsis we examined a number of factors 24 hours after LPS treatment. We found significant upregulation of F4/80 expressing cells with the expected hypertrophic activated microglial morphology, as well as increases in NOS2 and EGR-1 (Figure 10 and Figure S1). We did not observe changes in TNF-α or ARC in the SCN, nor were there any changes in the markers for apoptosis, TUNEL and cleaved casapse-3 (Figure 10).

## Discussion

The current study describes for the first time the long-lasting effects of sepsis on the mammalian circadian timing system. The findings that there are chronic effects of a single septic treatment with LPS are in agreement with findings that such treatment causes long-lasting effects on neuroinflammation and cognition in mice [22], [23], as well as with evidence that human survivors of sepsis suffer from long-term cognitive impairments [20]. To date there have been no comprehensive studies examining circadian parameters as output measures of post-septic encephalopathy. Our present findings indicate that by and large the circadian systems of post-septic mice do not differ greatly from controls: the core circadian parameters, such as free-running rhythm period and amplitude, or phase-angle of entrainment, are not altered following recovery from LPS treatment. For example, one might expect that if the central oscillator was weakened by LPS treatment then exposure to LL would result in either notably dampened rhythms or arrhythmicity. In fact neither is the case here, with rhythm robustness in LL similar in LPS-treated and control animals. Further, to demonstrate that entrainment is truly occurring under a L∶D cycle, and is not a result of masking in post-septic animals, we demonstrate that such animals entrain equally well as controls to skeleton photoperiods, where masking is not an issue due to the limited light exposure per day.

Differences were observed in post-septic animals in photic-induced phase advances, with such animals showing accelerated re-entrainment to advances of the L∶D cycle, and showing larger magnitude phase shifts in response to light pulses delivered in the late subjective night (but no changes at other phases). It is unclear by which mechanisms these changes may occur, although there are a number of possibilities, such as alterations in intracellular signalling pathways associated with phase advances but not delays (eg. the cGMP-PKG pathway [35]), or by alteration of glucocorticoid rhythms which in turn have been demonstrated to influence speed of re-entrainment [36]. Interestingly, in hamsters it has been shown that old age is associated with faster phase advances [37] and larger light pulse-induced phase advances [38]. Given that age is also associated with low-grade central neuroinflammation [39], including in the SCN [40], there may be a commonality of mechanism at this level with the LPS-induced microglial changes observed in the current study in the SCN. However, it should be noted that other studies of aging report slower re-entrainment or smaller phase shifts, and that other circadian changes associated with aging are not recapitulated in post-septic young animals (eg. dampened circadian amplitude [41]). Our finding that IEG responses to photic stimulation is not altered in the SCN argues that there are not gross problems in light-sensing or retinohypothalamic tract transmission to the SCN, but rather a more subtle mechanism is at play. These results are similar to those of Palomba and Bentivolgio [24] who report that weekly injection with 1 mg/kg LPS for 2 months induced impaired c-FOS induction by light in the early night phase 7 days after the last LPS treatment, but no differences 30 days after the last LPS treatment, a situation presumably more analogous to the experiments described here. It is also worth noting here that changes in SCN IEG expression do not always accompany changes in phase-shifting [42]. Further, the findings that phase shifts induced by a non-photic stimulus (8-OH-DPAT in this instance [28]) are similar between mice previously treated with LPS and controls suggests that augmentation of phase advances, irrespective of the stimulus, is not a feature of post-septic mice. However, it should be noted here that the phase-shifts elicited by 8-OH-DPAT in these experiments appear to be smaller than those reported by Horikawa and Shibata ([28]; 0.6–0.8 h reported here vs. ∼1 h for the previous report) and as such these results may be interpreted in the light of non-maximal responses not being sufficiently responsive to any influence of prior sepsis.

Alterations in clock gene expression are reported here in post-septic animals, with SCN expression of PER2 significantly altered with a lower expression at CT14 than in controls. Further to this, hippocampal expression of PER1 and PER2 is also altered, and given that the circadian control of hippocampal function appears to be dependent on SCN-derived signals [43], these alterations in the hippocampus may be a result of altered SCN output. It is interesting to note that the change in SCN PER2 expression is not translated into changes in free-running circadian parameters, and the seeming phase delay of the PER2 SCN rhythm in post-septic animals may be a result of the peak expression being dampened, rather than truly phase-shifted. Future experiments, for example using PER2::luc rhythms in the SCN *in vitro* derived from post-septic animals, should help resolve this. It is also not clear why there was a significant effect of prior sepsis on PER2, but not PER1 in the SCN, although modulation through different signalling mechanisms may be a factor here. There was a trend in our data towards PER1 suppression in post-septic animals at CT14, although this did not reach significance in our sample. A number of studies have shown that acute LPS treatment can alter clock gene expression. For example, in the rat SCN a septic dose of LPS suppressed levels of *per1* on the day of treatment but rhythmic expression returned on the day after treatment (although expression patterns were not followed for longer [44]). Acute treatment with lower doses of LPS has been shown to not alter *per1* in the mouse SCN [45] and the study of Guenther et al [46] shows no dampening effect of LPS treatment of SCN slice cultures on the rhythmic expression of the PER2::luc reporter. Clock gene expression in peripheral tissues has also been shown to be altered in response to LPS treatment (eg. heart and liver [47]; peripheral blood [48]). Further, a number of studies have demonstrated that pro-inflammatory mediators whose expression is upregulated by LPS can also impact on clock gene expression (eg. TNF-α [49], [50], [51]; IL-1β [49]; IFN-γ [18]; IL-6 [52]). Further, alterations in clock gene expression in the liver in response to CD40 stimulation were found to be dependent on the TNF-receptor 1 [53]. However, to the best of our knowledge, the current study is the first to describe chronic changes in clock gene expression patterns following a single septic dose of LPS (or following any other sepsis-inducing procedure). It is not clear at present how these alterations in clock gene expression patterns is brought about, although potential explanations include long-lasting changes in glial function that in turn may serve to modulate clock processes, long-term upregulation of neuroimmune mediators (although we find no such differences in factors such as TNF-α, IL-1 and NOS2 in the SCN in the present study), or alteration in endocrine function that in turn feeds back to the SCN (eg. glucocorticoid rhythms). All of these facets warrant future further investigation. Another important question is to whether changes in clock gene product expression in the post-septic SCN translate into functional differences in SCN state, and such questions may be addressed in future through the electrophysiological characterisation of SCN neurones in post-septic animals.

A number of previous studies have shown that acute LPS treatment can alter circadian/SCN function. Peripheral treatment with low dose LPS can elicit phase shifts when administered during the early night phase, with a concomitant increase in SCN c-FOS [15], and these LPS-induced effects appear to be dependent on TLR4 signalling [54]. Direct treatment of SCN slice cultures with LPS alters AVP production, indicating that the SCN may be capable of responding directly to LPS [55], suggesting that the SCN may express TLR4 and be sensitive directly to LPS. Septic doses of LPS have also been shown to acutely induce substantial c-FOS expression in the SCN, as well as activation of the p65 subunit of the NF-κB pathway [11], and these findings are in agreement with the current finding that 24 hours after septic LPS treatment there is a marked upregulation of SCN EGR-1. Following acute treatment with LPS we also observed an upregulation of F4/80 expressing microglia with activated morphology, again indicating that in the acute timescale the SCN responds to a substantial peripheral immune challenge. We examined ARC as chronic neuroinflammation induced by chronic i.c.v. infusion of LPS has been associated with an upregulation of ARC, an effector IEG rather than a transcription factor IEG like c-FOS and EGR-1, in the dentate gyrus [56]. However we found no evidence for either acute of chronic upregulation of ARC by LPS in the SCN.

We do not believe that the dose of LPS used in this study is inducing cell loss in the SCN, as we observed no changes in TUNEL or cleaved-caspase 3 staining during 24 hours following LPS treatment. This appears congruent to our findings that in the longer term the neuropeptides AVP and VIP were unaltered in the SCN, although levels of these are diminished in the aged SCN [57]. Weberpals et al [23] report that treatment of mice with the same dose of LPS used in the current study does not result in cell loss in the hippocampus or cerebral cortex, and Qin et al [22] show that only 7 months after LPS treatment was there neurodegeneration, which itself appeared to be confined to the substantia nigra. However as in the current study we have not assessed neuronal numbers, it is possible that there is cell loss through necrosis that is not detected by TUNEL/cleaved-caspase 3 assays. Another interesting finding from our study is that we do not observe changes, in either the short of the long term, in TNF-α expression in the SCN. Qin et al [22] report chronic upregulation of TNF-α in brain homogenates for many months following on from the same LPS treatment used in our study, although peripheral levels of TNF-α returned to baseline within 1 week of treatment. However, Weberpals et al [23] describe a regional variation in TNF-α and IL-1β upregulation in the long term following LPS, suggesting that the SCN may be a region that does not show such changes in TNF-α expression. Again similar to the findings of Weberpals et al study [23] we also do not see evidence for chronic astrocytic activation in the SCN, as evidenced by no changes in GFAP expression, but LPS-induced nitric oxide production via NOS2 may be important in eliciting changes in SCN function. Further, although previous evidence suggests that the NF-κB signalling pathway is co-opted in the SCN during the acute phase following LPS treatment [11], [58], our examination of three markers from this pathway (p-IκK, p-IκB, p65) does not indicate that the NF-κB signalling is chronically upregulated in the post-septic SCN, nor is there long-term upregulation of IL-1β in the SCN.

The relative lack of differences in the antigens examined between control and post-septic SCN suggests that other factors may be important in the long-lasting changes in circadian function observed. For example, given that septic doses of LPS causes chronic loss of cholinergic fibres in rats [59] and that cholinergic input to the SCN plays a role in circadian function [60], it would be of interest to examine cholinergic transmission in the post-septic SCN. It would be of further interest to examine the electrophysiological properties of SCN neurones following recovery from sepsis, and to further examine the neurochemical composition of the SCN in these animals (eg. are there changes in the GABAergic network as seen in the aged SCN [61]).

In conclusion, the current study illustrates for the first time the long-lasting effects of sepsis on circadian rhythms in mice, and further point to important influences between the circadian and immune systems. Given the roles of circadian rhythms in cognition [62] it may be that in human sepsis survivors that alterations of circadian function may be a factor in the cognitive difficulties that persist following recovery from the acute phase of septic shock.

## Supporting Information

Figure S1 **Photomicrographs illustrating SCN responses in the acute phase of sepsis, 24 following LPS treatment.** (A) shows the upregulation of F4/80 in hypertrophic microglia, (B) shows the increase in NOS2 expression and (B) shows the marked upregulation of EGR-1 in the SCN and peri-SCN/peri-ventricular region. Scale bar = 100 µm.
(DOCX)

Table S1 **Details of the antibodies used for immunohistochemistry in this study.**
(DOCX)
