# Supplementary figures and images for: Long-Lasting Effects of Sepsis on Circadian Rhythms in the Mouse
Source: PLoS One. 2012 Oct 11;7(10):e47087. doi: 10.1371/journal.pone.0047087 (PMC3469504; doi:10.1371/journal.pone.0047087)

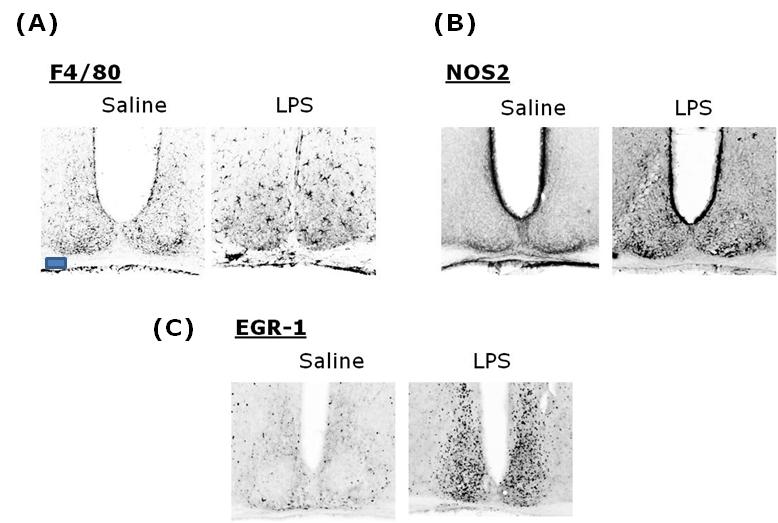

Supplement: Figure S1 — Photomicrographs illustrating SCN responses in the acute phase of sepsis, 24 following LPS treatment. (A) shows the upregulation of F4/80 in hypertrophic microglia, (B) shows the increase in NOS2 expression and (B) shows the marked upregulation of EGR-1 in the SCN and peri-SCN/peri-ventricular region. Scale bar = 100 µm. (DOCX) [file pone.0047087.s001.docx]
