# Supplementary material for: Long-Lasting Effects of Sepsis on Circadian Rhythms in the Mouse
Source: PLoS One. 2012 Oct 11;7(10):e47087. doi: 10.1371/journal.pone.0047087 (PMC3469504; doi:10.1371/journal.pone.0047087)
Supplement: Table S1 — Details of the antibodies used for immunohistochemistry in this study. (DOCX) [file pone.0047087.s002.docx]

| **Antibody** | **Dilution** | **Raised in** | **Supplier** | **Product Code** |
| --- | --- | --- | --- | --- |
| c-Fos (4) | 1:2000 | Rabbit | Santa Cruz Biotechnology, Germany | sc-52 |
| Arc (H-300) | 1:500 | Rabbit | Santa Cruz Biotechnology | sc-15325 |
| Egr-1 (C-19) | 1:3000 | Rabbit | Santa Cruz Biotechnology | sc-189 |
| CD11b | 1:1000 | Rat | AbD Serotec, Oxford, UK | MCA74GA |
| F4/80 | 1:100 | Rat | AbD Serotec, Oxford, UK | MCA497GA |
| GFAP | 1:1000 | Rabbit | Sigma, St. Louis, MO, U.S.A | G4546 |
| Cleaved Caspase-3 (Asp175) | 1:200 | Rabbit | Cell Signaling Technology, Danvers, MA, U.S.A. | #9661 |
| TNF- α | 1:75 | Rat | AbD Serotec, Oxford, UK | MCA1488 |
| IL-1β | 1:50 | Rabbit | Peprotech, U.S.A. | 500-P51 |
| NOS2 (M-19) | 1:100 | Rabbit | Santa Cruz Biotechnology | sc-650 |
| NFkB p65 (C-20) | 1:200 | Rabbit | Santa Cruz Biotechnology | sc-372 |
| p-IKK α/β (Ser 180/ Ser 181)-R | 1:200 | Rabbit | Santa Cruz Biotechnology | sc-23470-R |
| p-IkB- α (Ser32/36) | 1:200 | Rabbit | Santa Cruz Biotechnology | sc-101713 |
| AVP | 1:5000 | Rabbit | Millipore, Temecula, CA, U.S.A. | AB1565 |
| VIP | 1:1000 | Rabbit | Millipore, Temecula, CA, U.S.A. | AB982 |
| PER1(N20) | 1:500 | Goat | Santa Cruz Biotechnology | s-7724 |
| PER2 | 1:1000 | Rabbit | Alpha Diagnostics, Texas, USA | PER21-A |
| CLOCK (H-276) | 1:500 | Goat | Santa Cruz Biotechnology | sc-25361 |
